# Age, frequency of volunteering, and Present-Hedonistic time perspective predict donating items to people in need, but not money to combat COVID-19 during lock-down

**DOI:** 10.1007/s12144-021-01993-0

**Published:** 2021-06-23

**Authors:** Iwona Nowakowska

**Affiliations:** Institute of Psychology, The Maria Grzegorzewska University, Szczęśliwicka 40, 02-353 Warsaw, Poland

**Keywords:** Charitable behavior, COVID-19, Donating, Lock-down, Present-Hedonistic time perspective, Social value orientation, Time perspectives, Volunteerism, Young adults

## Abstract

Restrictions due to COVID-19 necessitated staying at home, but in some cases, encouraged charitable behavior, e.g., donating items to people in need (e.g., clothes, food), or money to support combatting COVID-19. Drawing on the previous findings regarding helping during disastrous situations and roles of time perspective in helping behaviors, the study tested the predictive value of age, gender, previous volunteering, altruistic social value orientation, and time perspectives of donating items to people in need or money to combat COVID-19. The study is pioneering in terms of including time perspectives as individual differences which might contribute to making donations during COVID-19 circumstances. The study was questionnaire-based and conducted online in the eighth week of social distancing in Poland. 150 young adults (age 18–35) took part in the study. Results of multivariable logistic regression analysis indicated that age, frequency of volunteering before the epidemic, and Present-Hedonistic time perspective predict donating items to people in need, but none of the tested variables predicted donating money to combat COVID-19. The findings suggest that charitable behavior, especially in the context of extraordinary social situations, needs to be treated as a multifaceted phenomenon. The study indicates that a Present-Hedonistic time perspective would be a promising individual difference to test in future studies on prosociality.

## Introduction

COVID-19 has changed the patterns of everyday functioning, causing governments worldwide to impose restrictions on social life, education, work, transportation, and travel (Zajenkowski et al., 2020). In many countries worldwide, the strict social distancing regulations included a lock-down-like measure – avoidance of any unnecessary social contact, as well as the obligation to wear masks in public spaces (Wang et al., 2020). The lock-down changed substantially the patterns of working (Dang et al., 2020; Tran et al., 2020), learning (Aristovnik et al., 2020; Zawadka et al., 2021), and spending leisure time (Bond et al., 2020) . It also had negative implications for mental health and distress (Gambin et al., 2021; Le et al., 2020; Wang et al., 2020; Wang et al., 2021).

Social support mobilization is typically observable during natural disasters (Kaniasty, 2020). Generally, people, as social creatures, have to cooperate and act prosocially to cope with the demands of the environment and changes that occur around them (Li et al., 2019). Prosocial behaviors, which are both intentional and voluntary, are aimed at benefitting another person (Lay & Hoppmann, 2015) and might include donating and sharing resources (Bartlett & DeSteno, 2006; Clark, 2005). It was observable in Poland that some people tried to deal with the situation of COVID-19 social distancing by connecting with others, either by seeking help or offering it. The lock-down encouraged the advent of social support networks that had not existed before, for instance, social media groups gathering people in preparation to help others in everyday activities (e.g., shopping, walking animals for quarantined people, donating clothes and food for people who had lost their jobs) (Jarynowski et al., 2020). These groups served not only to gather people interested in emergent informal volunteering (Whittaker et al., 2015) but also to be a platform of information exchange about people in need and about fundraising opportunities to support the state in combatting the COVID-19 crisis.

A review of correlates of philanthropy and generosity by Bekkers and Wiepking (2011) indicated that generosity in money donation tends to increase with age. Another review by Wiepking and Bekkers (2012) suggested that results on gender differences in philanthropic giving were mixed. In some studies, females were more inclined to give, whereas men tended to give higher amounts of money. However, researchers agree that sociodemographic features cannot fully explain people’s charitable behavior. The question is: what personal characteristics might be predictive of charitable behavior during the COVID-19 lock-down?

The current paper concentrates on two forms of charitable sharing: sharing items (e.g., clothes, food) with people in need and donating money to combat COVID-19. The research was conducted in the eighth week of lock-down in Poland, and started one day after the first compromises to the lock-down restrictions were introduced. The research question is whether the same hypothesized personal characteristics (frequency of volunteering before the epidemic, altruistic social value orientation, and time perspectives) that might be associated with prosocial behaviors are predictive of donating items to people in need during COVID-19 and to donating money to combat COVID-19.

### Charitable Behavior and Previous Volunteering

Charitable acts, including acts of generosity, might be associated with previous experiences with volunteering. Volunteering might be defined as devoting time to another person, group, or organization (Wilson, 2000). It is rarely a single act of kindness; it is often based on continuous prosocial engagement and requires devotion of time and effort for the benefit of people outside one’s own family. Volunteers tend to donate more (and more often) to charity than non-volunteers (Matsunaga, 2007). Other studies also suggest that the amount of time devoted to volunteering and donating to non-profit organizations have a positive relationship (Callen, 1994; Marcuello & Salas, 2001; Schervish & Havens, 1997).

Although studies suggest that the characteristics of a typical volunteer and a volunteer during a natural disaster are not the same (Michel, 2007). In extraordinary circumstances (for instance, September 11, 2001), voluntary support for victims of these circumstances is predicted by the individual’s engagement in volunteer work over the previous year (Steinberg & Rooney, 2005). Less is known about the predictors of generous sharing during such circumstances, however, it might be hypothesized that people who tend to engage in prosocial voluntary behaviors might be more inclined to share with others in need. The question is: during the unexpected, extraordinary circumstances of the COVID-19 lock-down, are people who engaged more frequently in volunteer activities before the epidemic more inclined to share items and money with others?

### Social Value Orientation, Prosocial Behaviors, and Donations

Social value orientation (SVO) is an individual difference in concern for other people (Murphy & Ackermann, 2014). It is often measured using a method of decomposed games, which are based on allocating resources between oneself and another person. Decomposed games are based on unilateral choices, in which the participant decides on both the amount of money (or other resources, e.g., points) he or she receives and the amount of money received by the other person. Most often, measures assessing SVO enable the classification of people into categories that describe the character of their social orientation: from highly competitive to highly altruistic. Van Lange et al. (2007) found that people who had a prosocial SVO engaged in a greater number of donations in real life, the recipients of which were mostly organizations supporting the ill and the poor. The result was understood as a form of enhancement of well-being motivated by looking for fairness and improving the outcomes of people who do not have as many resources as the donors. However, the study by Van Lange et al. (2007) was conducted comparing people classified as competitors, individuals, and prosocials, and the SVO was not treated as a continuous metric. The SVO Slider Measure (Murphy et al., 2011) is a metric that attempts to address the limitations of measures that do not enable differentiation between levels of altruistic orientation on a continuous scale. To date, such continuous metrics of SVO have rarely been employed to assess real-life generous acts. It is interesting to explore whether donating during the COVID-19 epidemic was associated with the level of altruistic SVO.

### Time Perspectives, Prosocial Behaviors, and Donations

An interesting individual difference, yet rarely investigated in terms of prosocial behavior, is time perspective. Time perspectives are individual differences in how people view their past, present, and future, and how these views affect their emotions and actions. It is a process of assignment of the flow of social and personal experiences to temporal categories to give coherence, meaning, and order to life events (Zimbardo & Boyd, 1999). Zimbardo and Boyd (1999, 2008) distinguished five time perspectives: Past-Negative, Past-Positive, Present-Fatalistic, Present-Hedonistic, and Future. Past time perspectives tend to predict emotional outcomes (Matthews & Stolarski, 2015; Nowakowska, 2020a), whereas present and future predict behaviors (Andre et al., 2018; Kooij et al., 2018; Przepiorka & Blachnio, 2016; Taquet et al., 2016).

Helping and other prosocial behaviors might also be predicted by individual differences in time perspectives. Zimbardo and Boyd (2008) claimed that future-oriented people tend to think so much about themselves and their personal goals that they do not engage in helping behaviors, in contrast to Present-Hedonistic-oriented people, whose concentration on the “here and now” serves as a positive predictor of prosocial behaviors. However, later considerations about the future time perspective broadened the understanding of it in the context of prosocial behaviors. Given that people high on future time perspective are capable of resisting the temptation to gain short-term benefits, and prosocial behavior might appear beneficial to them in the long run (Van Lange et al., 2013). Thus, consideration of such future benefits might encourage them to undertake such behaviors (Nostrand & Ojanen, 2018; van der Graaff et al., 2018). Maki et al. (2016) found that the future time perspective is connected to volunteerism and continuing it over time, and is associated with beliefs about volunteerism. In the same study, a momentary focus on the future (e.g., induced with a writing paradigm) was also linked to higher volunteering intentions, especially in people low on dispositional future time perspective and those who volunteer infrequently. Additionally, a study by Sjåstad (2019) suggested that people tend to be more generous in donations when focusing on the future, especially when the choice is framed publicly (when others will know the identity of the participant – donor). It suggests that the future time perspective activates donation intentions due to the reputational benefits it might bring.

Moreover, the Present-Fatalistic time perspective, although rarely explored in the context of prosocial behaviors, might be an interesting variable to test in the context of a lock-down. A recent study by Jimenez et al. (2020) suggested that fatalistic beliefs of COVID-19 as a death sentence were negatively associated with preventive behaviors taken to reduce the spread of the epidemic (e.g., handwashing, social distancing). Given that people who are Present-Fatalistic-oriented tend to think about their life as determined by fate and forces they cannot influence, their engagement in proactive behaviors directed at changing the status quo may be limited. Fatalism is also linked to hopelessness and lower self-efficacy (Straughan & Seow, 1998). Therefore, a higher Present-Fatalistic time perspective might be negatively associated with proactive coping by helping others during the lock-down.

Based on the abovementioned theoretical assumptions and findings, it seems particularly interesting to explore whether present and future time perspectives might be predictive of donations during an extraordinary circumstance – namely the COVID-19 lock-down.

### Current Study

The aim of this study is to find out whether the frequency of volunteering activities before the epidemic, altruistic SVO as well as Present-Fatalistic, Present-Hedonistic and Future time perspectives predict donation of items to people in need or donation of money during the COVID-19 lock-down, and whether the same predictors apply for these two forms of charitable behavior. Age and gender are controlled given their potential contribution to how people act charitably (Bekkers & Wiepking, 2011; Wiepking & Bekkers, 2012). The study goal is to broaden the scope of understanding donating behaviors during the epidemic by examining not only characteristics relevant to predict helping (frequency of volunteerism before COVID-19, altruistic SVO) but also time perspectives – a construct recently found out to be important for prosocial behaviors (Maki et al., 2016; Zimbardo & Boyd, 2008).

During emergencies and disasters, it is observed that not only professional, highly qualified workforces are helping people cope with the consequences of the extraordinary events. Ordinary citizens, for instance – informally volunteering to support others, are providing often fast support to people in need (Whittaker et al., 2015). Whittaker et al. (2015) proposed two broad types of volunteering during disasters: extending (when organizations extend their activities to meet needs observed during the disaster) and emergent (occurring in response to needs observed during the disasters). They also suggested that a novel mode of disaster volunteerism needs to be acknowledged – digital volunteerism, which might be both extending and emergence and which takes advantage of the availability and sophistication of online tools.

During COVID-19 in Poland, the digital mode of helping others played a vital part, especially in the strict lock-down and obligations to stay at home. It was observable especially in social media, where informal self-help groups named “Visible Hands” (Polish: “Widzialna Ręka”) were created (Jarynowski et al., 2020). Such groups were examples of emergent informal volunteering and more generally – emergent prosocial behavior, including donating. During the lock-down along with face-to-face helping, donations of items to people in need were relatively common in these groups. At the same time, several public fundraising opportunities were released there to support the state and the medical staff in combatting COVID-19. To date, much literature concentrated on volunteering during disasters, less – on donating. This research aims to fill this gap and find out whether donating items to people in need (helping persons) and donating money to combat COVID-19 (helping the society) can be predicted by the same personal characteristics.

Considering the theoretical assumptions and previous research findings, the following hypotheses were drawn:

- H1. *The frequency of volunteering will be positively associated with donating; however, the association will be stronger for donating items than donating money.* Volunteering is distinct from donating, but they are both forms of prosocial behavior. People who volunteered previously might be generally more inclined to help others and might have extended their activity during COVID-19 to donating, especially in lock-down, being unable to fulfill their commitment to an organization.
- H2. *A more altruistic SVO will be positively associated with donating.* SVO is measured as a tendency to altruistically share resources with others (Murphy et al., 2011). It is interesting whether this construct is related to real-life helping in disastrous situations such as lock-down due to COVID-19.
- H3. *Both Present-Hedonistic and Future time perspectives will be positively associated with donating.* Time perspectives have only recently been considered as valid predictors of helping behaviors (see Maki et al., 2016; Zimbardo & Boyd, 2008) and this sub-field of prosociality studies deserves further research. Present-Hedonistic-oriented people might be helpful to others since they intend to answer to needs “here and now” and seek pleasure from being supportive (Zimbardo & Boyd, 2008). It is also hypothesized that Future-oriented people might be helpful, given that long-term orientation to contribute to the well-being of the community after the disaster might promote providing support (Monllor et al., 2020). Future-oriented people might also tend to care for establishing and maintaining relationships with the help recipients which might be beneficial in the future (Chernyak-Hai & Halabi, 2018). Future time perspective also correlates with beliefs and behaviors regarding volunteerism (initiating and sustaining commitment) (Maki et al., 2016). Possibly also for donating behaviors such a pattern can be observed.
- H4. *A Present-Fatalistic time perspective will be negatively associated with donating.* Present-Fatalism has not been yet investigated in the context of helping during extraordinary circumstances. However, it might be predicted that given the nature of fatalism, people being high on the Present-Fatalistic time perspective might be less inclined to help others, not believing in the possibility to change the *status quo* and being generally less self-efficacious (Straughan & Seow, 1998).

## Method

### Procedure

The study was conducted online at the beginning of May 2020 – right after introducing the first phase of strict social distancing in Poland. The questions, therefore, referred to nearly two months of the strictest period of lock-down. The recruitment of participants took place on Facebook groups of higher education students and city groups, as well as on “Visible Hand” self-help groups (the features of these groups were discussed above). The questionnaires were gathered within one week to gather information for the defined context of the epidemic. The survey set was addressed to people between the age of 18 and 35 and was described as “a study of helping behaviors during COVID-19”. The study protocol was approved by the ethics committee at The Maria Grzegorzewska University. The study was anonymous and all participants provided informed consent to take part in the study. The participants were not remunerated.

### Measures

#### Donating Behaviors

The participants were directly asked how often they had donated items to people in need since the outbreak of the epidemic: one question was about donating directly, in person, and another about donating indirectly, e.g., through a volunteer. Another question asked whether they donated money to combat COVID-19. For the abovementioned questions, the participants marked their answers on an ordinal scale: 0 – never, 1 – once a month, 2–2-3 times a month, 3 – once a week, 4 – several times a week, 5 – every day.

#### Frequency of Volunteering before the Epidemic

The participants were directly asked how often they engaged in volunteering before the epidemic. They marked their answers on an ordinal scale: 0 – never in a lifetime, 1 – never in the last year, 2–1-2 times last year, 3 – several times last year, 4 – once a month last year, 5–2-3 times a month last year, 6 – once a week last year, 7 – more often than once a week in the last year.

#### Social Value Orientation

SVO was estimated with 6 primary items of Social Value Orientation Slider Measure Version A (Murphy et al., 2011; Polish version: Nowakowska, 2020b) and the algorithm of computing the SVO angle provided by Murphy et al., 2011 using a syntax by Baumgartner. The measure is a form of a decomposed game, where each of the items is a continuum of own/other payoff allocations. The payoff is described as money. The participant has to choose the most preferred joint outcomes. The items are designed so as to, using the algorithm, enable the results to locate the participant on one of the most commonly observed SVO types (altruistic, prosocial, individualistic, and competitive) based on cutoff points of the SVO angle, which is a continuous metric, taking negative and positive values. The SVO angle may take the lowest value of −16.26 (perfectly competitive) and the highest of 61.39 (perfectly altruistic). The higher the value of the angle, the more altruistic the SVO of the person. The measure has several strong points, such as a behavioral rather than a self-report character and it does not rely strongly on language. Moreover, it enables a continuous rather than only a categorical output to be employed, which offers the opportunity to analyze the degree of inclination toward altruism.

#### Time Perspectives

To measure time perspectives, the Zimbardo Time Perspective Inventory (ZTPI) was used (Zimbardo & Boyd, 1999; Polish version: Przepiórka, 2011). It is a 56-item tool, consisting of five subscales: Past-Negative (10 items, e.g., “I’ve taken my share of abuse and rejection in the past”), Past-Positive (9 items, e.g., “It gives me pleasure to think about my past”), Present-Fatalistic (9 items, e.g., “My life path is controlled by forces I cannot influence”), Present-Hedonistic (15 items, e.g., “Spending what I earn on pleasures today is better than saving for tomorrow’s security”) and Future (13 items, e.g., “I make lists of things to do”). For the purpose of the study, Present Fatalistic, Present-Hedonistic, and Future subscales were used. The participants score on a five-point scale reflecting the degree to which each statement refers to him/her (a range from 1 – very untrue to 5 – very true). Results in subscales were obtained by calculating mean scores from relevant items. Maximum 2 missing data were allowed for mean computation.

### Participants

A total of 150 participants aged 18–35 filled out the questionnaire set. 130 participants were female (86.7%), and 20 male (13.3%). The mean age of the participants was 25.72 (*SD* = 4.63). 30 (20.0%) of the participants lived in the country, 20 (13.3%) in a town of fewer than 100,000 inhabitants, 35 (23.3%) in a town of 100,001–499,999 inhabitants, and 65 (43.3%) in a town of over 500,000 inhabitants. 12 participants (8.0%) lived alone, and 138 (92.0%) with other people. 17 participants (11.3%) spent the lock-down period in one place with children, whereas 133 did not (88.7%). Education of participants was as follows: 1 (0.7%) – primary school, 2 (1.3%) – vocational school, 58 (38.7%) – high school, 41 (27.3%) – bachelor’s degree, 45 (30.0%) – Master’s degree, 3 (2.0%) – PhD degree or higher level of education.

During the COVID-19 lock-down, 20 participants (13.3%) worked only in their standard workplace, 16 (10.7%) remotely, but sometimes on-site, 37 (24.7%) only remotely, 15 (10.0%) had their job activity temporarily suspended, 13 (8.7%) had lost their job during lock-down, 43 (28.7%) did not work before the pandemic, and 6 (4.0%) declared other job status.

### Preliminary Analyses

All results within the subscales of the used measures are computed as means of items that correspond to subscales. In the case of the SVO angle, the result is a “SVO angle”, which is a continuous metric (Murphy et al., 2011); however, to avoid bias connected to the presence of negative numbers, the participants’ results were recoded by adding 16.26 to all participants’ results to obtain only a positive number output (the minimum SVO value possible is −16.26). This recoded value was saved as a separate variable and used for further analyses. It is important to acknowledge that due to this recoding, the results cannot be interpreted in terms of SVO types according to Murphy et al. (2011).

For dependent variables, data about the frequency of behavior were recoded into a dichotomous variable of donating items (directly or indirectly taken together) to people in need, or money to combat COVID-19: never during the COVID-19 lock-down (0, no) or at least once in this period of time (1, yes). Out of the 150 participants, 69 people at least once donated items to people in need during COVID-19 lock-down; 66 participants at least once donated money to combat COVID-19.

### Statistical Analysis

The analyses for this study were conducted with IBM SPSS 25.0.0.2 for Windows.

### Analytic Strategy

At first, descriptive statistics and rho-Spearman correlations between variables were computed. In the second step, relevant variables were entered as predictors in multivariate logistic regression for two models: one predicting donation of items during the COVID-19 lock-down and the other predicting donation of money to combat COVID-19 during the same period of time.

## Results

Taking into account the SVO types, based on the cutoff points by Murphy et al. (2011) and the non-recoded value of SVO angle, 1 participant was competitive (0.7%), 5 individualistic (3.3%), 139 prosocial (92.7%), and 5 altruistic (3.3%).

Table 1 presents the distribution of answers regarding the frequency of volunteering before the pandemic, setting of volunteering before the pandemic, duration of volunteer service, as well as frequencies of donations of items to people in need in person and through other people, as well as donations of money to combat COVID-19.

**Table 1:** Distribution of answers regarding volunteering before the pandemic and donations after its outbreak

	*N*	%
Frequency of volunteering before the pandemic		
Never in a lifetime	26	17.3
Never in the last year	42	28.0
1–2 times in the last year	28	18.7
A few times in the last year	22	14.7
Once a month in the last year	6	4.0
2–3 times a month in the last year	10	6.7
Once a week in the last year	6	4.0
More often than once a week in the last year	10	6.7
Setting of volunteering before the pandemic (multiple choice allowed)		
Medical	8	5.3
Education	33	22.0
Charity	51	34.0
Ecological organizations	7	4.7
Local community	36	24.0
Office	11	7.3
Culture	27	18.0
Work with animals	7	4.9
Other	7	4.9
Duration of volunteer service before the epidemic		
Less than a month	14	9.3
1–3 months	10	6.7
4–6 months	4	2.7
7–12 months	8	5.3
13–24 months	6	4.0
Over 24 months	47	31.3
Frequency of donating items to people in need directly (in person) since the outbreak of the pandemic		
Never	97	64.7
Once a month	36	24.0
2–3 times a month	11	7.3
Once a week	3	2.0
Several times a week	2	1.3
Every day	1	0.7
Frequency of donating items to people in need indirectly (e.g., through a volunteer) since the outbreak of the pandemic		
Never	109	72.7
Once a month	31	20.7
2–3 times a month	5	3.3
Once a week	4	2.7
Several times a week	0	0.0
Every day	1	0.7
Frequency of donating items to people in need indirectly (e.g., through a volunteer) since the outbreak of the pandemic		
Never	84	56.0
Once a month	42	28.0
2–3 times a month	22	14.7
Once a week	2	1.3

Table 2 presents correlations between continuous study variables, as well as between continuous and dichotomous study variables. It also presents means and standard deviations for continuous study variables.

**Table 2:** Rho-Spearman correlations between study variables

	1	2	3	4	5	6
1 Age	–					
2 Frequency of volunteering before the epidemic	.07	–				
3 SVO (recoded)	.09	.08	–			
4 Present-Fatalistic time perspective	−.19*	.00	−.08	–		
5 Present-Hedonistic time perspective	−.21**	−.04	.09	.25**	–	
6 Future time perspective	.17*	−.02	.01	−.40**	−.29**	–
*M*	25.72	–	53.58	2.62	3.22	3.52
*SD*	4.63	–	9.53	.62	.56	.58
*α*	–	–	–	.69	.79	.77

Note. * - *p* < .05, ** - *p* < .01

The correlation analysis indicated that age was negatively related to both Present-Fatalistic and Present-Hedonistic, and positively to Future time perspectives. Present-Fatalistic time perspective was positively correlated with Present-Hedonistic and negatively with Future time perspective. The Present-Hedonistic time perspective was also negatively related to the Future time perspective.

In the next step of the analysis, the relevant variables were entered into two models: one predicting donating items to strangers during COVID-19 (Model 1), and another predicting donating money to combat COVID-19 (Model 2). People who never engaged in these activities during lock-down were coded 0, people who did this at least once were coded 1. The results of multivariate logistic regression analysis are presented in Table 3. The models correctly classified 68.7% of cases (Model 1) and 58.7% of cases (Model 2).

**Table 3:** Multivariate logistic regression predicting donating: items to people in need during COVID-19 or money to combat COVID-19

Predictor	B	SE B	Wald	df	*p*	Exp(B)	95% CI
Model 1, χ^2^(7) = 22.73, *p* = .002, Nagelkerke = .188							
Gender	.18	.54	.11	1	.74	1.20	.42; 3.44
Age	.13	.04	9.40	1	<.01	1.14	1.05; 1.24
Frequency of volunteering before the epidemic	.23	.09	6.51	1	<.05	1.26	1.06; 1.50
SVO (recoded)	−.01	.02	.23	1	.64	.99	.96; 1.03
Present-Fatalistic time perspective	.26	.32	.68	1	.41	1.30	.70; 2.41
Present-Hedonistic time perspective	.90	.36	6.22	1	<.05	2.45	1.21; 4.94
Future time perspective	−.08	.35	.05	1	.83	.93	.47; 1.84
Model 2, χ^2^(7) = 4.71, *p* = .696, Nagelkerke = .041							
Gender	−.15	.51	.09	1	.77	.86	.32; 2.35
Age	.06	.04	2.70	1	.10	1.06	.99; 1.15
Frequency of volunteering before the epidemic	.05	.08	.36	1	.55	1.05	.89; 1.24
SVO (recoded)	.01	.02	.24	1	.62	1.01	.97; 1.05
Present-Fatalistic time perspective	.06	.30	.04	1	.85	1.06	.59; 1.90
Present-Hedonistic time perspective	.35	.33	1.17	1	.28	1.42	.75; 2.71
Future time perspective	.08	.32	.06	1	.81	1.08	.57; 2.04

Data from Table 3 suggests that the logistic regression model predicting donating items to strangers during COVID-19 was statistically significant, χ^2^(7) = 22.73, *p* < .01. The model explained 18.8% of the variance in donating items during COVID-19 (Nagelkerke *R*^2^). Older young adults were more likely to donate, as well as young adults who volunteered more frequently before the epidemic, and who had a more hedonistic time perspective. The same model for donating money to combat COVID-19 appeared to be non-significant χ^2^(7) = 4.71, *p* = .70; none of the variables predicted donating.

## Discussion

The goal of this study was to find out whether age, gender, frequency of volunteering activities before the epidemic, the level of altruistic SVO, and present or future time perspectives might predict donation of items to people in need, and money to combat COVID-19 during the lock-down.

None of the hypothesized variables were significant predictors of donating money to combat COVID-19. It is worth noting that it was the act of making a donation to combat COVID-19 that was taken into account and not the amounts that were donated. The coronavirus crisis might be understood as a strong social situation, highly structured and defined, and providing cues to guide behavior (Snyder & Ickes, 1985). The characteristics of strong social situations might be more prominent in predicting behaviors than personality traits (Sherman et al., 2012), e.g., traits that were investigated in the current study (time perspectives, altruistic social value orientation). However, it has to be noted that other personality traits (e.g., the Big Five or HEXACO personality traits) that were beyond the scope of the current study could have been predictive of donating money to combat COVID-19.

For donating items, age is positively related to displaying this behavior. This is in line with findings regarding the relationship between donating and age (Bekkers & Wiepking, 2011). The older the young adults, the more likely it is that they are more educated, have better-paid work, and have children – and therefore more resources (clothes, accessories) to be donated to other people in need.

Additionally, the frequency of volunteering predicted donating items to other people. Volunteering engagement was found to be associated with greater generosity (Matsunaga, 2007). As a sustained prosocial act, volunteering might be a form of realizing a person’s tendencies to help other people. Also, the lock-down might have limited the opportunities to volunteer in standard contexts of the organizations of origin. Therefore, volunteers might have sought alternative ways to fulfill their need to help others, and chose to, for instance, share resources with others.

Out of the investigated time perspectives, only Present-Hedonistic was related to donating items during the COVID-19 lock-down. It is in line with Zimbardo and Boyd’s (2008) assumption that being in the “here and now” facilitates concern about the current situation and needs of the other and promotes prosocial action. The present study was conducted in Poland, which was, according to an international study, a country in which depressive, anxiety, and stress symptoms were relatively high in the general population (Wang et al., 2021). The Present-Hedonistic time perspective was proven to predict a higher level of depressive symptoms in men and a lower level of depressive symptoms in women during the first lock-down in spring 2020 (Bodecka et al., 2021). Therefore, this time perspective might be hypothesized to play a role in how people experienced distress during the social distancing period. It is worth noting that the result in a study by Bodecka et al. (2021) was obtained in a similar period to the current study. The present study adds to this finding, suggesting that people who donated items to others were also higher on the Present-Hedonistic time perspective. The sample consisted mainly of women (*N* = 130, 86.7% of the sample). Therefore, sharing with others might have been a protective factor from experiencing distress during lock-down or a form of coping and reducing negative feelings by distraction from concerns about the situation in the country and/or physical sensations which might have been indicative of contracting the disease (for a discussion of the interplay of need for health information, physical symptoms, and mental health see Wang et al., 2021).

The Present-Hedonistic time perspective is also linked to impulsivity (Jochemczyk et al., 2017; Zimbardo & Boyd, 1999) and might promote both maladaptive and adaptive forms of coping (Blomgren et al., 2016). Through an attitude of concentrating on the present moment, Present Hedonists might have reacted impulsively, not concentrating on accumulating resources for oneself, but sharing them instead. Prosocial sharing with others might also promote satisfaction and happiness (Aknin et al., 2011) and therefore Present-Hedonistic oriented people, who typically seek pleasure in the moment might have coped with the situation of the lock-down and uncertainty through prosocial action. This is an interesting result which opens up new research avenues on hedonism as a promoter of prosocial action.

Prosocial behavior might be driven by both egoistic and altruistic motivations (Lay & Hoppmann, 2015). True altruism is when an act is driven by a desire to benefit the other without own benefits (Eisenberg & Miller, 1987; Feigin et al., 2014). Egoistic motivations include, e.g., the desire to feel better about oneself, improve own reputation and avoid negative feelings (Feigin et al., 2014; Penner et al., 2005). If the hedonistic motivation of seeking pleasure from helping is prominent, the actions cannot be considered truly altruistic. This found its confirmation in the lack of association between donating and SVO. Moreover, a recent study by Jasielska and Rajchert (2020) suggested a lack of significant correlation between SVO and communion orientation and a significant negative correlation with agency. Thus, also in the current study, SVO could have been non-useful in predicting outcomes that are related to serving the community and which might at the same time require a high sense of agency (such as engagement in donations during the COVID-19 lock-down).

Participation of citizens is crucial in reducing disaster consequences and supporting resilience (Whittaker et al., 2015) and stands in contrast to the directive risk management provided by professional services (Wiek et al., 2010). Prosocial behavior brings people together and enhances productive and peaceful coexistence (Lay & Hoppmann, 2015). It also enables people to feel part of a wider community (Bai et al., 2017; Piff et al., 2015), which might have been very supportive during the uncertain time of lock-down.

### Limitations and Future Research Directions

Several limitations of this study need to be taken into account when interpreting the findings. First of all, the sample consisted of mainly females, therefore the results cannot be considered representative for the population of young adults. Moreover, the frequency of volunteering before the epidemic was measured on an ordinal scale, which does not provide full information about the frequency of volunteering. Such a limitation can be overcome in future studies by measuring the frequency of such behavior on an interval scale (e.g., the average number of days of volunteering in a month). Furthermore, the model for donating items to people in need during the epidemic, although proving significant associations between the dependent variable and age, frequency of volunteering, and Present-Hedonistic time perspective accounted for 18.8% of the variance in donating items during the COVID-19 lock-down. It is worth including additional variables in future studies, such as the Big Five personality traits or personal values, to find out whether predictors that turned out to be significant would remain predictive of donating items.

The study was conducted at the time of the first lock-down when apprehension regarding the virus was mixed with a lack of knowledge about it. Donating items means passing things between people, and that requires hygiene and sanitization of the items, and the knowledge about this important matter has been growing in time with the spread of the pandemic (Nguyen et al., 2021). The question is, therefore, whether after a year of the pandemic people are still willing to share items, and how fear of the coronavirus and/or being vaccinated or not impact the readiness to share material resources with other people.

The pandemic and the requirement to remain socially distanced changed the way how prosociality can express itself (Albarracín & Jung, 2021). The readiness to adhere to COVID-19-related preventive measures are linked to prosocial concerns (Pfattheicher et al., 2020), similarly – the readiness to vaccinate (Chew et al., 2021; Jung & Albarracín, 2021). Given that the topic is highly relevant for global safety and health, it is worth further investigating to find out what factors can contribute to acting for the long-term collective interest instead of focusing on narrow self-interest (Johnson et al., 2020). The current study indicates that time perspectives could be a potentially interesting research avenue in this field.

The current study was cross-sectional and conclusions cannot be derived on the sustainment of donating behaviors after the lock-down. The participants were also recruited online through randomly selected groups, therefore the data cannot be considered representative of the population. It is worthwhile to continue studies on donating patterns after the epidemic to find out whether the observed social support mobilization is only temporary and connected to the lock-down context or connected to the personal characteristics of participants.

## References

[CR1] Aknin, L. B., Sandstrom, G. M., Dunn, E. W., & Norton, M. I. (2011). Investing in others: Prosocial spending for (pro)social change. In R. Biswas-Diener (Ed.), *Positive psychology as social change* (pp. 219–234). Springer. 10.1007/978-90-481-9938-9_13.

[CR2] Albarracín D, Jung H (2021). A research agenda for the post-COVID-19 world: Theory and research in social psychology. Asian Journal of Social Psychology.

[CR3] Andre L, van Vianen AE, Peetsma TT, Oort FJ (2018). Motivational power of future time perspective: Meta-analyses in education, work, and health. PLoS One.

[CR4] Aristovnik A, Keržič D, Ravšelj D, Tomaževič N, Umek L (2020). Impacts of the COVID-19 pandemic on life of higher education students: A global perspective. Sustainability.

[CR5] Bai Y, Maruskin LA, Chen S, Gordon AM, Stellar JE, Mcneil GD (2017). Awe, the diminished delf, and collective engagement: Universals and cultural variations in the small self. Journal of Personality and Social Psychology.

[CR6] Bartlett MY, DeSteno D (2006). Gratitude and prosocial behavior: Helping when it costs you. Psychological Science.

[CR7] Bekkers R, Wiepking P (2011). Who gives? A literature review of predictors of charitable giving part one: Religion, education, age and socialisation. Voluntary Sector Review.

[CR8] Blomgren AS, Svahn K, Åström E, Rönnlund M (2016). Coping strategies in late adolescence: Relationships to parental attachment and time perspective. The Journal of Genetic Psychology.

[CR9] Bodecka M, Nowakowska I, Zajenkowska A, Rajchert J, Kaźmierczak I, Jelonkiewicz I (2021). Gender as a moderator between present-hedonistic time perspective and depressive symptoms or stress during COVID-19 lock-down. Personality and Individual Differences.

[CR10] Bond AJ, Widdop P, Cockayne D, Parnell D (2020). Prosumption, networks and value during a global pandemic: Lockdown leisure and COVID-19. Leisure Sciences.

[CR11] Callen JL (1994). Money donations, volunteering and organizational efficiency. Journal of Productivity Analysis.

[CR12] Chernyak‐Hai, L., & Halabi, S. (2018). Future time perspective and interpersonal empathy: Implications for preferring autonomy‐versus dependency‐oriented helping. *British Journal of Social Psychology, 57*(4), 793–814. 10.1111/bjso.12260 10.1111/bjso.12260 29926924

[CR13] Chew NW, Cheong C, Kong G, Phua K, Ngiam JN, Tan BY (2021). An Asia-Pacific study on healthcare worker’s perception and willingness to receive COVID-19 vaccination. International Journal of Infectious Diseases.

[CR14] Clark N (2005). Disaster and generosity. The Geographical Journal.

[CR15] Dang AK, Le XTT, Le HT, Tran BX, Do TTT, Phan HTB (2020). Evidence of COVID-19 impacts on occupations during the first Vietnamese national lockdown. Annals of Global Health.

[CR16] Eisenberg N, Miller PA (1987). The relation of empathy to prosocial and related behaviors. Psychological Bulletin.

[CR17] Feigin S, Owens G, Goodyear-Smith F (2014). Theories of human altruism: A systematic review. Journal of Psychiatry and Brain Functions.

[CR18] Gambin, M., Sękowski, M., Woźniak-Prus, M., Wnuk, A., Oleksy, T., Cudo, A., ... & Maison, D. (2021). Generalized anxiety and depressive symptoms in various age groups during the COVID-19 lockdown in Poland. Specific predictors and differences in symptoms severity. *Comprehensive Psychiatry, 105*, 152222. 10.1016/j.comppsych.2020.152222 10.1016/j.comppsych.2020.152222 33388494

[CR19] Jarynowski A, Wójta-Kempa M, Płatek D, Czopek K (2020). Attempt to understand public health relevant social dimensions of COVID-19 outbreak in Poland. Society Register.

[CR20] Jasielska D, Rajchert J (2020). When is happy also prosocial? The relationship between happiness and social orientation depends on trust, agency and communion. Current Issues in Personality Psychology.

[CR21] Jimenez T, Restar A, Helm PJ, Cross RI, Barath D, Arndt J (2020). Fatalism in the context of COVID-19: Perceiving coronavirus as a death sentence predicts reluctance to perform recommended preventative behaviors. SSM – Population Health.

[CR22] Jochemczyk Ł, Pietrzak J, Buczkowski R, Stolarski M, Markiewicz Ł (2017). You only live once: Present-hedonistic time perspective predicts risk propensity. Personality and Individual Differences.

[CR23] Johnson, T., Dawes, C., Fowler, J., & Smirnov, O. (2020). Slowing COVID-19 transmission as a social dilemma: Lessons for government officials from interdisciplinary research on cooperation. *Journal of Behavioral Public Administration, 3*(1). 10.30636/jbpa.31.150.

[CR24] Jung H, Albarracín D (2021). Concerns for others increases the likelihood of vaccination against influenza and COVID-19 more in sparsely rather than densely populated areas. Proceedings of the National Academy of Sciences.

[CR25] Kaniasty K (2020). Social support, interpersonal, and community dynamics following disasters caused by natural hazards. Current Opinion in Psychology.

[CR26] Kooij DT, Kanfer R, Betts M, Rudolph CW (2018). Future time perspective: A systematic review and meta-analysis. Journal of Applied Psychology.

[CR27] Lay JC, Hoppmann CA (2015). Altruism and prosocial behavior. Encyclopedia of Geropsychology.

[CR28] Le HT, Lai AJX, Sun J, Hoang MT, Vu LG, Pham HQ (2020). Anxiety and depression among people under the nationwide partial lockdown of Vietnam. Frontiers in Public Health.

[CR29] Li JJ, Dou K, Wang YJ, Nie YG (2019). Why awe promotes prosocial behaviors? The mediating effects of future time perspective and self-transcendence meaning of life. Frontiers in Psychology.

[CR30] Maki A, Dwyer PC, Snyder M (2016). Time perspective and volunteerism: The importance of focusing on the future. The Journal of Social Psychology.

[CR31] Marcuello C, Salas V (2001). Nonprofit organizations, monopolistic competition, and private donations: Evidence from Spain. Public Finance Review.

[CR32] Matsunaga Y (2007). To give, or not to give; to volunteer, or not to volunteer, that is the question: Evidence on Japanese philanthropic behavior revealed by the JGSS-2005 data set. JGSS Research Series.

[CR33] Matthews, G., & Stolarski, M. (2015). Emotional processes in development and dynamics of individual time perspective. In M. Stolarski, N. Fieulaine, & P. G. Zimbardo (Eds.), *Time perspective theory; review, research and application* (pp. 269–286). Springer. 10.1007/978-3-319-07368-2_18.

[CR34] Michel LM (2007). Personal responsibility and volunteering after a natural disaster: The case of hurricane Katrina. Sociological Spectrum.

[CR35] Monllor J, Pavez I, Pareti S (2020). Understanding informal volunteer behavior for fast and resilient disaster recovery: An application of entrepreneurial effectuation theory. Disaster Prevention and Management: An International Journal.

[CR36] Murphy RO, Ackermann KA (2014). Social value orientation: Theoretical and measurement issues in the study of social preferences. Personality and Social Psychology Review.

[CR37] Murphy, R. O., Ackermann, K. A., & Handgraaf, M. (2011). Measuring social value orientation. *Judgment and Decision Making, 6*(8), 771–781. 10.2139/ssrn.1804189.

[CR38] Nguyen, T. H. T., Le, H. T., Le, X. T. T., Do, T. T. T., Van Ngo, T., Phan, H. T., et al. (2021). Interdisciplinary assessment of hygiene practices in multiple locations: Implications for COVID-19 pandemic preparedness in Vietnam. *Frontiers in Public Health, 8*, 589183. 10.3389/fpubh.2020.589183. 10.3389/fpubh.2020.589183 PMC7870988 33575240

[CR39] Nostrand FV, Ojanen T (2018). Forms of prosocial behaviors are differentially linked to social goals and peer status in adolescents. The Journal of Genetic Psychology.

[CR40] Nowakowska, I. (2020a). Lonely and thinking about the past: The role of time perspectives, Big Five traits and perceived social support in loneliness of young adults during COVID-19 social distancing. *Current Issues in Personality Psychology, 8*(3), 175–184. 10.5114/cipp.2020.97289.

[CR41] Nowakowska, I. (2020b). Social Value Orientation Slider measure by Murphy et al. (2011). Instruction in Polish. Open Science Framework. 10.17605/OSF.IO/BRDQ7.

[CR42] Penner LA, Dovidio JF, Piliavin JA, Schroeder DA (2005). Prosocial behavior: Multilevel perspectives. Annual Review of Psychology.

[CR43] Pfattheicher S, Nockur L, Böhm R, Sassenrath C, Petersen MB (2020). The emotional path to action: Empathy promotes physical distancing and wearing of face masks during the COVID-19 pandemic. Psychological Science.

[CR44] Piff PK, Dietze P, Feinberg M, Stancato DM, Keltner D (2015). Awe, the small self, and prosocial behavior. Journal of Personality and Social Psychology.

[CR45] Przepiórka, A. (2011). *Kwestionariusz ZTPI* [Zimbardo Time Perspective Inventory questionnaire]. Retrieved from: http://www.timeperspective.net/uploads/2/5/4/4/25443041/polish.pdf. Accessed 10th Aug 2020.

[CR46] Przepiorka A, Blachnio A (2016). Time perspective in internet and Facebook addiction. Computers in Human Behavior.

[CR47] Schervish PG, Havens JJ (1997). Social participation and charitable giving: A multivariate analysis. Voluntas: International Journal of Voluntary and Nonprofit Organizations.

[CR48] Sherman RA, Nave CS, Funder DC (2012). Properties of persons and situations related to overall and distinctive personality-behavior congruence. Journal of Research in Personality.

[CR49] Sjåstad H (2019). Short-sighted greed? Focusing on the future promotes reputation-based generosity. Judgment and Decision making.

[CR50] Snyder, M., & Ickes, W. (1985). Personality and social behavior. In G. Lindzey & E. Aronson (Eds.), *Handbook of social psychology* (Vol. 2, 3rd ed., pp. 883–947). Random House.

[CR51] Steinberg KS, Rooney PM (2005). America gives: A survey of Americans’ generosity after September 11. Nonprofit and Voluntary Sector Quarterly.

[CR52] Straughan PT, Seow A (1998). Fatalism reconceptualized: A concept to predict health screening behavior. Journal of Gender, Culture and Health.

[CR53] Taquet M, Quoidbach J, De Montjoye YA, Desseilles M, Gross JJ (2016). Hedonism and the choice of everyday activities. Proceedings of the National Academy of Sciences.

[CR54] Tran BX, Nguyen HT, Le HT, Latkin CA, Pham HQ, Vu LG (2020). Impact of COVID-19 on economic well-being and quality of life of the Vietnamese during the national social distancing. Frontiers in Psychology.

[CR55] Van der Graaff J, Carlo G, Crocetti E, Koot HM, Branje S (2018). Prosocial behavior in adolescence: Gender differences in development and links with empathy. Journal of Youth and Adolescence.

[CR56] Van Lange PA, Bekkers R, Schuyt TN, Vugt MV (2007). From games to giving: Social value orientation predicts donations to noble causes. Basic and Applied Social Psychology.

[CR57] Van Lange PA, Joireman J, Parks CD, Van Dijk E (2013). The psychology of social dilemmas: A review. Organizational Behavior and Human Decision Processes.

[CR58] Wang C, Chudzicka-Czupała A, Grabowski D, Pan R, Adamus K, Wan X, Hetnał M, Tan Y, Olszewska-Guizzo A, Xu L, McIntyre RS, Quek J, Ho R, Ho C (2020). The association between physical and mental health and face mask use during the COVID-19 pandemic: A comparison of two countries with different views and practices. Frontiers in Psychiatry.

[CR59] Wang C, Chudzicka-Czupała A, Tee ML, Núñez MIL, Tripp C, Fardin MA, Habib HA, Tran BX, Adamus K, Anlacan J, García MEA, Grabowski D, Hussain S, Hoang MT, Hetnał M, le XT, Ma W, Pham HQ, Reyes PWC, Shirazi M, Tan Y, Tee CA, Xu L, Xu Z, Vu GT, Zhou D, Chan NA, Kuruchittham V, McIntyre RS, Ho CSH, Ho R, Sears SF (2021). A chain mediation model on COVID-19 symptoms and mental health outcomes in Americans, Asians and Europeans. Scientific Reports.

[CR60] Whittaker J, McLennan B, Handmer J (2015). A review of informal volunteerism in emergencies and disasters: Definition, opportunities and challenges. International Journal of Disaster Risk Reduction.

[CR61] Wiek A, Ries R, Thabrew L, Brundiers K, Wickramasinghe A (2010). Challenges of sustainable recovery processes in tsunami affected communities. Disaster Prevention and Management: An International Journal.

[CR62] Wiepking P, Bekkers R (2012). Who gives? A literature review of predictors of charitable giving. Part two: Gender, family composition and income. Voluntary Sector Review.

[CR63] Wilson J (2000). Volunteering. Annual Review of Sociology.

[CR64] Zajenkowski M, Jonason PK, Leniarska M, Kozakiewicz Z (2020). Who complies with the restrictions to reduce the spread of COVID-19?: Personality and perceptions of the COVID-19 situation. Personality and Individual Differences.

[CR65] Zawadka, J., Miękisz, A., Nowakowska, I., Plewko, J., Kochańska, M., & Haman, E. (2021). Remote learning among students with and without reading difficulties during the initial stages of the COVID-19 pandemic. *Education and Information Technologies*. 10.1007/s10639-021-10559-3. 10.1007/s10639-021-10559-3 PMC8068561 33935575

[CR66] Zimbardo, P., & Boyd, J. (2008). *The time paradox*. Free Press.

[CR67] Zimbardo PG, Boyd JN (1999). Putting time in perspective: A valid reliable individual differences metric. Journal of Personality and Social Psychology.

